# Mechanical thrombectomy for acute ischemic stroke after cardiac surgery or intervention: a retrospective cohort analysis

**DOI:** 10.3389/fneur.2025.1705053

**Published:** 2026-02-05

**Authors:** Rosa M. Eckert, Sarah Zweynert, Constanze Czimmeck, Maximilian Schoels, Georg Bohner, Eberhard Siebert, Yuriy Hrytsyna, Evgenij Potapov, Volkmar Falk, Christoph J. Ploner, Jörg Brandes, Christoph Leithner

**Affiliations:** 1 Department of Neurology and Experimental Neurology, Charité – Universitätsmedizin Berlin, Corporate Member of Freie Universität Berlin and Humboldt-Universität zu Berlin, Berlin, Germany; 2 Berlin Institute of Health (BIH), Berlin, Germany; 3 Institute of Neuroradiology, Charité – Universitätsmedizin Berlin, Corporate Member of Freie Universität Berlin and Humboldt-Universität zu Berlin, Berlin, Germany; 4 Department of Cardiothoracic and Vascular Surgery, Deutsches Herzzentrum der Charité (DHZC), Berlin, Germany; 5 DZHK (German Center for Cardiovascular Research), Partner Site Berlin, Berlin, Germany; 6 Department of Health Sciences and Technology, ETH Zurich, Zurich, Switzerland

**Keywords:** cardiac intervention, cardiac surgery, large vessel occlusion, stroke, thrombectomy

## Abstract

**Background:**

Acute stroke due to large vessel occlusion (LVO) is a serious complication of cardiac surgery or other cardiac interventions. Little is known about the epidemiological characteristics of affected patients, the temporal relationship between stroke detection and surgery/intervention, the efficacy of mechanical thrombectomy, or the associated clinical outcomes.

**Methods:**

We retrospectively analyzed the demographic and thrombectomy characteristics and neurological outcomes of patients who underwent mechanical thrombectomy for acute ischemic stroke due to LVO after cardiac surgery/intervention in a large academic heart center.

**Results:**

From January 2018 to January 2022, a total of 39 patients underwent thrombectomy for acute ischemic stroke with LVO following cardiac surgery/intervention. The median age was 66 years (IQR 57.5–76.0), and 13 patients (33.3%) were female. The highest frequency of thrombectomy for LVO-related stroke was observed after left ventricular assist device (LVAD) surgery (1.9%), followed by coronary artery bypass grafting (CABG) (0.20%), transcatheter aortic valve replacement (0.14%), and heart catheterization (0.04%). Stroke symptoms were detected in a wake-up constellation in 20 of the 39 patients (51.3%). Successful recanalization (TICI 2b/3) was achieved in 83.8% of patients. At three months, 21.2% of patients attained a good functional outcome (modified Rankin scale score 0–2).

**Conclusions:**

Thrombectomy for LVO stroke was conducted in a small subset of patients after cardiac surgery/intervention. A large proportion of these strokes were detected in a wake-up constellation. Early detection, optimized acute neurological workup, and rapid thrombectomy may result in good functional outcomes. The establishment of a standardized diagnostic and treatment algorithm seems advisable for the optimization of acute stroke treatment in large heart centers.

## 1 Introduction

Ischemic stroke is a severe complication of cardiac surgery or other cardiovascular interventions. The incidence varies according to surgery/intervention and patient cohort: 1.3%−2.1% after coronary artery bypass graft (CABG), 1.4%−4.3% after transcatheter aortic valve replacement (TAVR), 0.2%−0.96% after percutaneous coronary intervention, and up to 16.4% after left ventricular assist device (LVAD) implantation (1–10). Strokes following cardiac surgery/intervention are associated with high morbidity and mortality (1, 11, 12). In a typical stroke cohort, 10%−20% of all ischemic strokes are due to large vessel occlusions (LVOs) (13). A recent systematic review and meta-analysis identified only six observational studies involving a total of 72 patients who underwent thrombectomy after cardiac surgery/intervention (9, 14–18). A large diagnostic and procedure code database analysis identified 194 cases of thrombectomy for LVO stroke among 12,093 patients (1.6%) with prior cardiac surgery/intervention (19); however, individual patient characteristics could not be assessed in that study.

Thus, there is a knowledge gap regarding the frequency of thrombectomy for LVO stroke in relation to specific cardiac surgeries/interventions, the distribution of cerebral artery occlusion sites, the severity of acute ischemia, the timing of symptom detection, the success rate of recanalization, and the resulting functional outcomes. To address this, we performed a retrospective observational cohort study of consecutive patients who underwent thrombectomy following cardiac surgery/intervention, using data from a prospectively maintained thrombectomy database at a large academic heart center.

## 2 Materials and methods

### 2.1 Study design

The study was approved by the local ethics committee of Charité – Universitätsmedizin Berlin (Ethikkommission der Charité, approval number: EA2/121/22). The requirement for informed consent was waived as the study involved a retrospective analysis of routine clinical data. The Strengthening the Reporting of Observational studies in Epidemiology (STROBE) guidelines were followed (Supplementary material). Full access to all the data and full responsibility for its integrity and analysis were held by RE, SZ, and CL. The anonymized data used in this analysis are available from the corresponding author upon reasonable request.

A prospectively designed database of mechanical thrombectomies (MT) performed between January 2018 and January 2022 in patients treated at Deutsches Herzzentrum Berlin (DHZB), known as Deutsches Herzzentrum der Charité (DHZC) since the beginning of 2023, was retrospectively reviewed. All patients who underwent MT for acute stroke with LVO within 30 days after cardiac surgery/intervention were included in the study.

### 2.2 Baseline, stroke, imaging, and thrombectomy characteristics

The baseline demographic and clinical data collected comprised age, sex, cardiac disease leading to hospitalization, pre-existing conditions (arterial hypertension, diabetes mellitus, atrial fibrillation, dyslipidemia, smoking status, previous stroke), and type of cardiac surgery/intervention before stroke. The following stroke characteristics were assessed: time of symptom onset/symptom detection in relation to last surgery/intervention, ongoing sedation at the time of symptom detection, time of extubation, wake-up constellation, main neurological symptoms, National Institutes of Health Stroke Scale (NIHSS) score, and modified Rankin Scale (mRS) score (20–22). In case of wake-up constellation, last seen well (LSW) and time of symptom detection were evaluated. If the time of LSW was only specified as “before start of surgery/intervention,” the time point of intubation was recorded as LSW. The total number of surgeries/interventions was determined from administrative records.

The modality used for acute and follow-up imaging, the location of the LVO, and whether intracranial hemorrhage was present upon follow-up were recorded. The success of thrombectomy was evaluated according to the Thrombolysis in Cerebral Infarction (TICI) score (23). Thrombectomy devices used were recorded and categorized into “aspiration catheter,” “stent-retriever,” and “aspiration catheter and stent-retriever combined.” Time points of groin puncture for mechanical thrombectomy, additional endovascular treatments, and complications were assessed.

### 2.3 Outcome evaluation

The mRS score at discharge and at three months was determined from medical records, including neurological consultations, discharge summaries, rehabilitation reports, and documentation from neurological or other follow-up appointments. Scores were estimated from available clinical and neurological status documentation by two authors (SZ and RE). The scoring was determined by consensus and, in cases of disagreement, resolved through discussion guided by the more experienced examiner (SZ). If no data were available for the three-months point post-stroke, the mRS score was determined based on the neurological status at the two closest time points before and after three months. The mRS score at three months was judged unavailable if no neurological status was known later than 80 days after stroke. A favorable outcome was defined as a mRS score of 0–2 at three months. Outcomes at three months were compared between wake-up and non-wake-up patients, patients with and without ongoing sedation at the time of symptom detection and patients still intubated vs. those extubated at the time of symptom detection. Causes of death were evaluated, if possible, from available medical records and classified as “stroke” vs. “cardiac” vs. “other.”

### 2.4 Statistical analysis

Statistical analysis was performed using Microsoft Excel (version 16.0) and MATLAB (version R2021b). Categorical variables are reported as absolute numbers and percentages. Continuous data are summarized as medians with interquartile ranges (IQR).

## 3 Results

### 3.1 Epidemiological and stroke characteristics

A total of 39 patients who underwent thrombectomy for acute stroke with LVO after cardiac surgery/intervention between January 2018 and January 2022 were included. The median age was 66 years (IQR 58–76), and 13 patients were female (33%). Stroke was most commonly detected on the first post-operative day (IQR 0–2 days). In more than half of the patients (*n* = 20; 51%), stroke symptoms were detected in a wake-up constellation. The median NIHSS score was 14 (IQR 10–19), and the median mRS score was 5 (IQR 4–5) at first consultation. The characteristics of all the patients are summarized in Table 1.

**Table 1:** Demographic and stroke characteristics.

**Epidemiological and stroke characteristics (*n* = 39)**	**Absolute number of patients/median (percentage/IQR)**
Age, years, median (IQR)	66 (57.5–76.0)
Female sex, *n* (%)	13 (33.3)
Length of hospital stay, days, median (IQR)	17 (4.5–29.5)
**Pre-existing conditions**, ***n*** **(%)**	
Arterial hypertension	27 (69.2)
Diabetes mellitus	16 (41.0)
Atrial fibrillation	20 (51.3)
Dyslipidaemia	20 (51.3)
(Former) smoker	17 (43.6)
Previous stroke	8 (20.5)
**Stroke characteristics**	
Wake-up stroke, *n* (%)	20 (51.3)
POD of stroke, median (IQR)	1 (0–2)
NIHSS score before thrombectomy, median (IQR)^a^	14 (10–19)
mRS score before thrombectomy, median (IQR)^b^	5 (4, 5)

POD, postoperative day; IQR, interquartile range; NIHSS, National Institutes of Stroke Health Scale; mRS modified Rankin Scale.

^a^Data missing for four patients.

^b^Data missing for three patients.

### 3.2 Type of cardiac surgery/intervention before stroke

The frequencies of MTs for the individual surgeries/interventions are presented in Table 2. Twenty-six patients (66.7%) underwent a single surgery/intervention before the acute stroke. Of this patient subgroup, six (15.4%) received LVAD surgery (implantation or pump replacement), five (12.8%) underwent coronary bypass grafting, five (12.8%) underwent cardiac catheterization, and three (7.7%) received TAVR. Seven patients (17.9%) received other interventions. In one-third of the patients (*n* = 13; 33.3%), at least two procedures were combined during pre-stroke surgery/intervention. The individual surgeries/interventions, the cardiac diseases leading to hospitalization, and the stroke symptoms are listed in Supplemental Table 1.

**Table 2:** Frequency of mechanical thrombectomy per type of surgery/intervention.

**Type of surgery/intervention**	**Number of surgeries/interventions**	**Number of MTs (rate per surgery/intervention, %)**
LVAD	575	11 (1.91)
CABG	4,101	10 (0.24)
Aortic surgery (aorta replacement)	2,065	4 (0.19)
Valve surgery/intervention	6,413	8 (0.12)
TAVR	2,175	3 (0.14)
Cardiac catheterization	15,481	6 (0.04)

Note that in cases of combined surgeries/interventions, one MT patient appears in both groups, and thus the total number of MTs listed in this table exceeds the total number of MTs performed in the entire cohort.

MT, mechanical thrombectomy; LVAD, left ventricular assist device; CABG, coronary artery bypass graft; TAVR, transcatheter aortic valve replacement.

### 3.3 Stroke symptoms

Overall, hemiparesis was present in 29 patients (74.4%) at first consultation. Impaired consciousness was detected in 13 (33.3%) patients, aphasia and/or dysarthria in 10 (25.6%) patients, pupil dysfunction or an oculomotor disorder in 6 (15.4%) patients and visual impairment in 2 (5.1%) patients.

### 3.4 Stroke imaging

The characteristics of stroke imaging are shown in Table 3. All 39 patients received head non-contrast computed tomography (NCCT) plus computed tomography angiography (CTA), while 4 (10.3%) were additionally subjected to computed tomography perfusion (CTP) imaging. LVO was detected in 37 cases (94.9%), and symptomatic high-grade stenosis without complete occlusion was identified in two patients (5.1%).

**Table 3:** Stroke imaging characteristics.

**Imaging characteristics of all patients (*n* = 39)**	**Absolute number of patients (percentage)**
**Acute imaging**, ***n*** **(%)**	**39 (100)**
Type of imaging, *n* (%)	
NCCT + CTA	35 (89.7)
NCCT + CTA + CTP	4 (10.3)
Early signs of ischemia, *n* (%)	2 (5)
Infarct demarcation, *n* (%)	13 (33.3)
Chronic infarction, *n* (%)	10 (25.6)
Vessel occlusion, *n* (%)	37 (94.9)
Intracerebral hemorrhage, *n* (%)	0
**Follow-up imaging**, ***n*** **(%)**	**39 (100)**
Type of imaging, *n* (%)	
MRI	2 (5.1)
NCCT	26 (66.7)
NCCT + CTA	10 (25.6)
NCCT + CTA + CTP	1 (2.6)

NCCT, non-contrast computed tomography; CTP, computed tomography perfusion; CTA, computed tomography angiogram; MRI, magnetic resonance imaging.

### 3.5 Location of vessel occlusion and thrombectomy characteristics

The location of vessel occlusion and thrombectomy characteristics for all 39 patients are portrayed in Table 4. Bridging thrombolysis was performed in three patients (7.7%). Anterior circulation occlusions predominated, with almost half of the occlusions affecting the first segment of the middle cerebral artery, the intracranial carotid artery, or the second segment of the middle cerebral artery (M2). Technically successful recanalization (TICI 2b/3) was achieved in 31 patients (83.8%). Aspiration catheters and stent-retrievers combined were used for recanalization in most patients (*n* = 22, 56.4%). Aspiration catheters only were used in eight patients (20.5%), stent retrievers only in three (7.7%).

**Table 4:** Location of vessel occlusion and mechanical thrombectomy characteristics.

Additional intravenous tPA (bridging thrombolysis), *n* (%)	3 (7.7)
Technically successful recanalization (TICI score 2b/3), *n* (%)^a, b^	31 (83.8)
Number of passes, median (IQR)	1 (1–3)
**Type of vessel occlusion**, ***n*** **(%)**	
**Single vessel occlusion**	
ICA	4 (10.3)
Carotid T	1 (2.6)
M1	12 (30.8)
M2	7 (17.9)
BA	4 (10.3)
**Multiple vessel occlusions**	
M1 and M2	3 (7.7)
M2 and A1	1 (2.6)
All supraaortal large arteries	1 (2.6)
P1 and ICA	1 (2.6)
M1 and ICA	1 (2.6)
PCA on both sides	2 (5.1)
**Others**	
High-grade M1 stenosis	1 (2.6)
High-grade ICA stenosis	1 (2.6)
**Side of vessel occlusion**, ***n*** **(%)**	
Right	16 (41.0)
Left	16 (41.0)
Bilateral	2 (5.1)
Not applicable	5 (12.8)
Additional symptomatic extracranial ICA stenosis, *n* (%)	4 (10.3)

tPA, tissue plasminogen activator; TICI, Thrombolysis in Cerebral Infarction; IQR, interquartile range; ICA, internal carotid artery; M1, first segment of the middle cerebral artery, right after the ICA; M2, second, more distal segment of the middle cerebral artery; BA, basilar artery; A1, first segment of the anterior cerebral artery, right after the ICA; A4 fourth segment of the anterior cerebral artery; P1, first segment of the posterior cerebral artery, right after the BA; PCA, posterior cerebral artery; DHZC, Deutsches Herzzentrum der Charité.

^a^Referring to the major occlusion side.

^b^No intervention needed for two patients.

A detailed analysis of additional endovascular treatments and complications of mechanical thrombectomy is provided in Supplemental Table 2. Two patients (5.1%) suffered vascular perforation, eight patients (20.1%) experienced vasospasm, clot migration/a new ischemic event in a new vascular territory/a new vascular occlusion was observed in three patients (7.7%) and four patients (10.3%) experienced subarachnoid hemorrhage after thrombectomy.

No complications associated with intravenous tPA in addition to thrombectomy were observed among the three patients (7.7%) who underwent bridging thrombolysis.

### 3.6 Processing times

Median time of symptom detection to groin puncture was 2 h and 12 min (1:36–2:55, IQR) in patients awake at the time of symptom detection and 2 h and 44 min (1:55–4:20, IQR) in wake-up stroke patients. For the 20 patients with wake-up strokes, time of last seen well (LSW) and time of symptom detection were documented in 19 (95%). The median time between LSW and time of symptom detection in these patients was 11 h (IQR: 6–25 h).

### 3.7 Outcome

**Figure 1:**
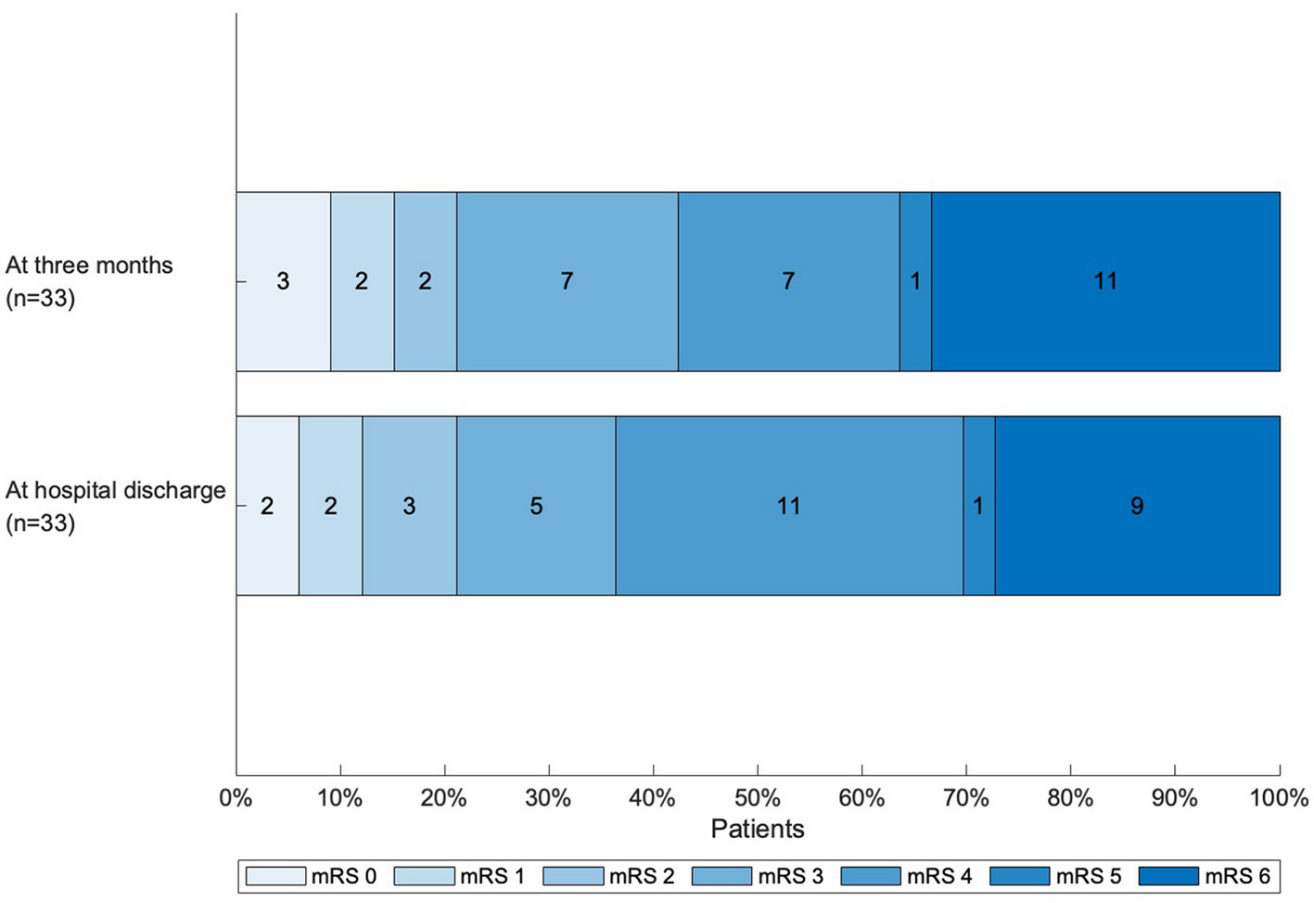
Functional outcome at hospital discharge and at 3 months, as determined by the mRS score (patients for whom there was no data at 3 months were excluded). mRS, modified Rankin scale.

For all 39 patients, functional outcome at hospital discharge was assessed using medical records. Three-month outcome was available for 33 patients (84.6%). Due to the retrospective nature of our study, three-months outcome was unavailable for six patients. Figure 1 illustrates outcomes according to the mRS score at hospital discharge and at three months. At discharge, seven patients had a favorable outcome (mRS score 0–2; 17.9%), and the median mRS score was 4 (IQR 3–6). Nine patients (23.1%) died during their hospital stay. At three months, seven patients (21.2%) had a favorable outcome, while 11 (33.3%) had died. Disagreements of scoring occurred in three patients (7.7 % at discharge, 9% at 3 months). Of the 11 patients who died, four died of stroke (36.4%), two of cardiac causes (18.2%) and one of other causes (9%). For four patients, causes of death could not be determined with certainty.

Outcomes at three months according to clinical characteristics at time of symptom detection are provided as Supplemental Table 3. Favorable outcome was more frequent for patients with symptoms detected awake [6/19 patients (32%) with mRS 0–2] as compared to wake-up stroke patients [1/20 (5%) with mRS 0–2]. Similarly, outcome was better for patients off sedation at the time of symptom detection as compared to those still sedated and better for patients extubated as compared to those still intubated at the time of symptom detection. Due to the low number of patients in these subgroups, these differences should be interpreted cautiously. Of note, a significant number of patients in all subgroups survived with mRS 3–4 at 3 months.

## 4 Discussion

Our main findings were as follows: (i) thrombectomy for acute stroke with LVO was performed at a low but clinically relevant frequency in a large academic heart center; (ii) the risk for stroke due to LVO was especially high in LVAD surgeries; (iii) in half of the cohort, stroke symptoms due to LVO were detected upon awakening; (iv) a relevant subgroup of patients (21.2%) had a favorable functional outcome (mRS score 0–2) at three months after MT; (v) a significant proportion of patients experienced a poor outcome or death within three months of the stroke despite technically successful recanalization; and (vi) in our cohort, one quarter of patients presented with multivessel occlusions.

Over 4 years, 39 patients with acute ischemic stroke due to LVO received thrombectomy in a large academic heart center in which more than 37,000 cardiac surgeries/interventions were performed during the same period. This corresponds to a frequency of 0.10% acute strokes due to LVO per surgery/intervention, comparable to the 0.11% reported by Sheriff et al. (17) and the 0.08% reported by Wilkinson et al. (18). Approximately one thrombectomy was performed per month.

In a recent analysis of a large diagnostic and procedure code database, de Havenon et al. (19) found that out of 12,093 patients with a diagnosis of stroke and a cardiac surgery/intervention preceding the stroke, 194 underwent a MT, corresponding to only 1.6% of stroke cases. Using a similar approach, Alkhouli et al. reported that thrombectomy was performed in 4.4% of patients with stroke after TAVR (47). In contrast, an international multicenter registry documented that 6.7% of all patients with stroke after TAVR received thrombectomy (9). In general stroke populations, 10%−20% of all strokes are due to LVO (13). These findings indicate that earlier detection of LVO after cardiac surgery/intervention, combined with rapid diagnosis and treatment, could increase the use of thrombectomy. In line with this interpretation, Sheriff et al. (17) and Wilkinson et al. (18) both reported rates of 9%−11% for thrombectomy for LVO in patients with acute stroke after cardiac surgery/intervention.

We found large differences in thrombectomy rates for individual cardiac surgeries/interventions. The risk was highest for LVAD surgeries, followed by extracorporeal membrane oxygenation/intra-aortic balloon pump implantation, and was considerably lower for coronary bypass grafting, valve surgeries, TAVR, and heart catheterization. This aligns well with the stroke rates of up to 16% reported for LVAD surgeries vs. approximately 2% for CABG and TAVR and 0.5% for heart catheterization (1–9). Consistent with observations in general stroke populations, approximately 10% of our cohort underwent MT for stroke after cardiac surgery/intervention (24). These numbers may be valuable for estimating the expected burden of acute LVO strokes and subsequent MTs in cardiac centers.

Studies on general ischemic stroke populations reported wake-up stroke rates between 8 and 28%, considerably lower than the 51% recorded in our cohort (25, 26). This high rate in our cohort reflects the timing of stroke detection in relation to surgery/intervention. Most strokes occurred within 2 days (median day 1, IQR 0–2), close to the day 0 timepoint reported in a recent review and meta-analysis (14). The most likely explanation for the high incidence of wake-up strokes in our study is the post-operative setting, with many strokes occurring during ongoing anesthesia or sedation. Importantly, findings from the DAWN and DEFUSE-3 randomized controlled trials demonstrated a substantial benefit of thrombectomy in patients with wake-up or later-presenting strokes (27, 28). These trials provided a strong rationale for administering MT in post-operative stroke patients with LVO. Subsequent trials have expanded the indication for thrombectomy to include patients with large infarct core volumes (29–31). Consequently, implementing standards for the timely detection of neurological deficits in cardiac surgery/intervention patients is important for reducing onset-to-treatment times and improving outcomes (32, 33). Potential approaches include non-invasive ICU monitoring techniques (e.g., electroencephalography, near infrared spectroscopy, somatosensory evoked potentials, arm accelerometry, video surveillance with artificial intelligence analysis), bedside clinical evaluation tools, and strategies for rapid sedation reduction (34–40).

The clinical benefit of bridging thrombolysis prior to thrombectomy in patients with acute stroke due to LVO remains an open question. Some studies found no difference between this combined approach and thrombectomy alone (41–44). In our study, only three of the 39 patients received bridging thrombolysis due to contraindications (recent surgery/intervention) and elapsed time windows. This pattern mirrors those of other studies, in which most patients similarly did not receive additional recombinant tissue plasminogen activator (9, 15, 16, 18).

Data on functional outcome for patients receiving thrombectomy for LVO after cardiac surgery/intervention is sparse. A recent systematic review and meta-analysis found that 42.7% of patients had a good functional outcome after thrombectomy following cardiac surgery/intervention (13). However, this high figure was likely attributable to a reporting bias. The reported proportions of good outcomes and mortality have differed widely between individual studies (15–18). For instance, in general stroke populations, good functional outcomes (mRS score 0–2) have shown considerable variation (14%−49%), as have mortality rates (14%−38%) at 90 days (27–31, 45). In comparison, in our cohort, the proportion of patients with good functional outcome was relatively low (21%), while the mortality rate was relatively high (33%), likely explained by the severe cardiac comorbidity of this population, particularly among patients with LVAD implantation.

We observed a numerically lower rate of good outcomes in wake-up stroke patients after cardiac surgery/intervention as compared to patients already awake at the time of symptom detection. In our post-operative/intervention cohort, last-seen well was at a median of 11 h before symptom detection while symptom detection to groin puncture times was around 2–3 h in both groups of severely ill cardiac surgery/intervention patients with complex logistics for thrombectomy. Although this data has to be interpreted cautiously due to the low number of patients in the subgroups, our observation is in line with literature on general stroke populations (46) and is likely related to the longer ischemia times in wake-up patients. Our preliminary finding of poorer outcome in wake-up stroke patients after cardiac surgery/intervention underscores the need to develop effective early cerebral ischemia recognition in these patients, e.g., via early wake-up calls and/or non-invasive brain monitoring devices.

Our study showed that stroke with LVO is a relevant clinical problem in large cardiac centers. Stroke with LVO requiring rapid MT may occur at a frequency of close to one case per month. Over recent years, therapeutic options for stroke with LVO have expanded considerably, particularly regarding treatment within extended time windows or the treatment of patients with large core infarcts. In parallel, diagnostic evaluation has become increasingly complex. As outcomes depend largely on rapid recanalization, the implementation of interdisciplinary diagnostic and treatment algorithms for stroke patients in cardiac centers seems advisable. This includes instructions for advanced cerebral imaging (CTA, CT-perfusion with mismatch volume determination) and precisely structured logistics for rapid diagnosis and treatment initiation.

### 4.1 Strengths and limitations

Our study is the largest in-depth analysis to date of individual patient data on acute ischemic stroke due to LVO treated with MT after cardiac surgery/intervention. Although our analysis was retrospective, patients were identified from a prospectively maintained thrombectomy database, thereby minimizing the risk of selection bias. However, patients with acute ischemic stroke due to LVO who did not undergo MT were not registered in this database. An analysis of this group of patients would have been valuable for identifying barriers to early stroke detection and developing more efficient strategies for early stroke recognition. Furthermore, when not documented, the mRS/NIHSS scores had to be estimated from the patients' records. Although neurologists were involved in the care of all patients and detailed neurological examinations were available, the estimation of these scores from the clinical records may not have been accurate in every case. Despite being the largest analysis of its kind, our sample size is relatively small. This limits the precision of estimates of rates and times, particularly in our subgroups. In line with many large stroke studies, we decided to determine outcome at three months (27, 28). A shift from the poor to the good outcome group seems possible, especially for patients with mRS 3–4 at three months. Finally, our study was monocentric and conducted at a large academic heart center. Importantly, the characteristics of patients and surgeries/interventions, the technical equipment used, and the experience of the cardiology, cardiac surgery, neurology, and interventional neuroradiology teams may differ across institutions, which limits the generalizability of our findings.

## 5 Conclusion

Thrombectomies for acute strokes with LVO occur at a clinically relevant frequency after cardiac surgeries/interventions in large cardiac centers. Stroke symptoms are frequently detected in a wake-up constellation. Good functional outcomes can be achieved when rapid thrombectomy is performed. Accordingly, we recommend the implementation of interdisciplinary, consensus algorithms for early diagnosis and rapid treatment, thus increasing the likelihood of a good functional outcome. Despite optimal treatment, a considerable proportion of patients will continue to experience poor outcomes. Larger, prospective multicenter studies are necessary for a more precise estimate of LVO stroke/MT frequencies in individual surgeries/interventions and outcome frequencies in patient subgroups.

## Data Availability

The raw data supporting the conclusions of this article will be made available by the authors, without undue reservation.
