# Supplementary material for: Mechanical thrombectomy for acute ischemic stroke after cardiac surgery or intervention: a retrospective cohort analysis
Source: Front Neurol. 2026 Feb 5;16:1705053. doi: 10.3389/fneur.2025.1705053 (PMC12916410; doi:10.3389/fneur.2025.1705053)
Supplement: Supplementary file 1 [file Data_Sheet_1.pdf]

Supplementary Material

1 Supplementary Tables

Supplemental table 1 Cardiac diagnosis, surgery/intervention and main symptom of stroke of 39 patients receiving mechanical thrombectomy for acute stroke with LVO

Patient no	Cardiological main diagnosis which led to the last cardiac surgery/intervention	Last cardiac surgery/intervention (before first consultation)	Main symptom of stroke
**1**	ascending aortic aneurysm AND 1-vessel CHD AND ramus intermedius stenosis	ascending aorta replacement AND CABG (1) AND PFO closure AND following rethoracotomy (bleeding)	right-sided hemiparesis AND conjugate eye deviation to the left side
**2**	3-vessel CHD AND angina pectoris CCS3 AND ejection fraction 50%	CABG (2)	left-sided hemiparesis
**3**	aortic valve stenosis AND aortic valve insufficiency AND 3-vessel CHD	AVR AND mitral valve decalcification AND bypass grafting	pupil dysfunction AND left-sided hemiparesis
**4**	mixed aortic valve disease with aortic stenosis and insufficiency AND mitral valve insufficiency AND tricuspid valve insufficiency AND atrial septal defect AND ascending aortic aneurysm AND pulmonary hypertension	AVR AND mitral annular decalcification AND ascending aorta replacement AND ASD closure AND LAA and RAA closure AND maze procedure AND VAC placement	left-sided hemiplegia
**5**	cable infection (LVAD)	VAC replacement (wound cable infection LVAD)	seizure

**6**	3-vessel CHD AND aortic valve stenosis	AVR AND aortocoronary venous bypass grafting	conjugate eye deviation to the left side AND vigilance impairment
**7**	atrial fibrillation	cardiac catheterization (isthmus ablation of atrial fibrillation)	right-sided hemiparesis AND aphasia
**8**	defect cable (LVAD)	LVAD pump replacement	aphasia AND right-sided hemiparesis
**9**	aortic valve stenosis	TAVR	vigilance impairment AND left-sided hemiparesis
**10**	CHD AND atrial fibrillation	cardiac catheterization AND cardioversion of atrial fibrillation	right-sided hemiparesis AND aphasia
**11**	intermittent third-degree atrioventricular heart block	dual chamber pacemaker implantation	vigilance impairment AND anisocoria AND conjugate eye deviation to the left side
**12**	3-vessel CHD (infarction)	CABG (3) AND IABP implantation	vigilance impairment
**13**	Aortic valve stenosis AND 3-vessel CHD	CABG bypass grafting AND AVR	vigilance impairment AND dysarthria
**14**	aortic aneurysm AND aortic valvular insufficiency	Ascending aorta replacement AND proximal aortic arch replacement AND aortic valve relocalization	aphasia AND right-sided arm paresis
**15**	decompensated ischemic cardiomyopathy (caused by 3-vessel CHD)	LVAD implantation AND impella explantation	vigilance impairment
**16**	obstructive and haemorrhagic shock (perforation of RAA caused by	surgical suture RAA AND epicardial ablation AND LAA clip	left-sided hemiplegia AND aphasia

	electrophysiology study of the heart)		
**17**	dilated cardiomyopathy (of undetermined origin) AND severe heart insufficiency	LVAD implantation	left-sided hemiparesis AND vigilance impairment
**18**	aortic valvular insufficiency AND ascending aortic aneurysm	AVR AND ascending aorta replacement	anisocoria
**19**	Impella heart pump thrombosis AND heparin-induced thrombocytopenia type 2	impella replacement	right-sided hemiparesis AND vigilance impairment
**20**	preparation for operation --> planned ascending aortic replacement	cardiac catheterization	left-sided hemiparesis
**21**	elective admission (coronary angiography)	coronary angiography	left-sided hemiparesis
**22**	dilated cardiomyopathy AND cardiac shock	LVAD implantation (additional peri-interventional "Backwash" manoeuvre)	eye rolling AND bilateral tonic-clonic myoclonus
**23**	3-vessel CHD	CABG bypass grafting (3 - a. and v.)	right-sided brachiofacial hemiparesis
**24**	3-vessel CHD	duplex ultrasonography of arm vessels (preoperative)	aphasia AND right-sided hemiplegia
**25**	emergency admission --> acute cardiac decompensation and acute pulmonary oedema caused by dilated cardiomyopathy	right cardiac catheterization	vigilance impairment AND right-sided hemiparesis
**26**	poor wound healing (wound impella heart pump implantation)	wound revision AND VAC placement (Re-LVAD implantation)	aphasia AND right-sided hemiparesis

**27**	CHD AND ischemic cardiomyopathy AND left ventricular recovery	impella explantation	left-sided hemiparesis AND dysarthria
**28**	dilated cardiomyopathy (status post myocarditis) AND cardiac shock	LVAD implantation	right-sided hemiparesis
**29**	3-vessel CHD	CABG bypass grafting (1)	vigilance impairment
**30**	3-vessel CHD (recent infarction)	CABG (3)	left-sided hemiparesis AND vigilance impairment
**31**	mixed aortic valve disease with aortic stenosis and insufficiency	TAVR	right-sided hemiparesis AND vigilance impairment
**32**	3-vessel CHD AND heart insufficiency	cardiac catheterization	dysarthria AND left-sided hemiparesis
**33**	3-vessel CHD (recent infarction)	CABG (3 vessels)	left-sided hemiparesis AND vigilance impairment
**34**	outflow graft stenosis	stenting of outflow graft stenosis of LVAD	facial paresis
**35**	aortic valve stenosis	TAVR	blindness
**36**	dilated cardiomyopathy AND terminal heart insufficiency (Low cardiac output syndrome (LCOS))	LVAD implantation	right-sided hemiparesis
**37**	dilated cardiomyopathy AND terminal heart insufficiency	LVAD implantation	left-sided hemiparesis
**38**	terminal heart insufficiency caused by ischemic cardiomyopathy	LVAD implantation AND impella explantation	left-sided hemiplegia

**39**	cardiac conduction disorder (atrial fibrillation, third-degree atrioventricular heart block)	dual chamber pacemaker implantation	left-sided hemiplegia AND aphasia AND visual impairment

CHD coronary heart disease, *CCS* Canadian Cardiovascular Society Score for angina pectoris, *LVAD* left ventricular assist device, *RAA* right atrial appendage *CABG* coronary artery bypass grafting, *PFO* patent foramen ovale, *AVR* aortic valve replacement, *LAA* left atrial appendage, *VAC* vacuum assisted closure-therapy, *TAVR* transcatheter aortic valve replacement, *IABP* intra-aortic balloon pump

Supplemental table 2 Complications and additional treatment of mechanical thrombectomy patients

**Mechanical thrombectomy characteristics [a]**	**DHZC patients (n=39)**
**Complications, n (%)**	
**Vascular perforation**	2 (5.1)
**Vasospasm**	8 (20.5)
**Clot migration/New ischemic event in a new vascular territory/New occlusion**	3 (7.7)
**SAH**	4 (10.3)
**Persistent occlusion after thrombectomy [b]**	10 (25.6)
**Additional treatment, n (%)**	
**ICA Balloon PTA**	2 (5.1)
**ICA stenting**	3 (7.7)
**Stenting of other vessels**	1 (2.6)
**Ballon PTA of other vessels**	1 (2.6)

SAH subarachnoid haemorrhage. *PTA* percutaneous transluminal angioplasty, *ICA* internal carotid artery, *DHZC* Deutsches Herzzentrum der Charité
[a] multiple answers per patient are possible

[b] distant vessels, not accessible for further thrombectomy attempts (M2 in one case, M3/4 in five cases, A1 in one case, A4 in one case, P3 in one case)

Supplemental table 3 Outcome at three-months for wake-up strokes vs. non-wake-up strokes, patients with ongoing sedation at symptom detection vs. patients off sedation at symptom detection and extubated vs. still intubated at symptom detection vs. never intubated patients

	**mRS 0**	**mRS 1**	**mRS 2**	**mRS 3**	**mRS 4**	**mRS 5**	**mRS 6**	**Median symptom detection to groin puncture (hours: minutes, IQR)**
**Wake-Up stroke patients (n=20)**	0	0	1	2	4	1	7	2:44 (1:55-04:20)
**Non-wake-up stroke patients (n=19)**	3	2	1	5	3	0	4	2:12 (1:36-2:55) [e]
**Ongoing sedation (n=13) [a]**	1	0	1	1	4	1	4	3:24 (2:40-06:34) [f]
**Off sedation (n=26) [b]**	2	2	1	6	3	0	7	2:09 (1:50-2:41) [g]
**Extubated (n=13)**	1	0	1	4	2	0	5	2:42 (2:13-3:00) [h]
**Still intubated (n=13) [c]**	1	0	0	0	4	1	4	2:40 (1:15-8:39) [i]

**Never intubated (n=13)** [d]	1	2	1	3	1	0	2	1:55 (1:33-2:20)

mRS modified Rankin Scale, *LSW* Last seen well, *IQR* Inter quartile range

[a] *one outcome is missing*
[b] *five outcomes are missing*
[c] *three outcomes were missing*
[d] *three outcomes were missing*
[e] *time of symptom detection to groin puncture (non-wake-up) two were missing*
[f] *time of symptom detection to groin puncture (Ongoing sedation) one was missing*
[g] *time of symptom detection to groin puncture (off sedation) one was missing*
[h] *time of symptom detection to groin puncture (extubated) two were missing*
[i] *time of symptom detection to groin puncture (still intubated) one was missing*

2 Supplementary Guideline

STROBE Statement—checklist of items that should be included in reports of observational studies

	Item No	Recommendation	Section
**Title and abstract**	1	(*a*) Indicate the study's design with a commonly used term in the title or the abstract	Title page
		(*b*) Provide in the abstract an informative and balanced summary of what was done and what was found	Title page
**Introduction**			
Background/rationale	2	Explain the scientific background and rationale for the investigation being reported	Introduction
Objectives	3	State specific objectives, including any prespecified hypotheses	Introduction
**Methods**			
Study design	4	Present key elements of study design early in the paper	Materials and Methods – Study design

Setting	5	Describe the setting, locations, and relevant dates, including periods of recruitment, exposure, follow-up, and data collection	Materials and methods
Participants	6	(*a*) *Cohort study*—Give the eligibility criteria, and the sources and methods of selection of participants. Describe methods of follow-up	Materials and methods
Variables	7	Clearly define all outcomes, exposures, predictors, potential confounders, and effect modifiers. Give diagnostic criteria, if applicable	Materials and methods – Outcome evaluation
Data sources/ measurement	8*	For each variable of interest, give sources of data and details of methods of assessment (measurement). Describe comparability of assessment methods if there is more than one group	Materials and methods
Bias	9	Describe any efforts to address potential sources of bias	Materials and methods – Outcome evaluation
Study size	10	Explain how the study size was arrived at	Materials and methods – Study design
Quantitative variables	11	Explain how quantitative variables were handled in the analyses. If applicable, describe which groupings were chosen and why	Materials and methods
Statistical methods	12	(*a*) Describe all statistical methods, including those used to control for confounding	Materials and methods – Statistical analysis
		(*b*) Describe any methods used to examine subgroups and interactions	Materials and methods – Statistical analysis
		(*c*) Explain how missing data were addressed	Materials and methods
		(*d*) *Cohort study*—If applicable, explain how loss to follow-up was addressed	Statistical analysis – Outcome evaluation
		(*e*) Describe any sensitivity analyses	n.a

**Results**			
Participants	13*	(a) Report numbers of individuals at each stage of study—eg numbers potentially eligible, examined for eligibility, confirmed eligible, included in the study, completing follow-up, and analysed	Results
		(b) Give reasons for non-participation at each stage	n.a
		(c) Consider use of a flow diagram	n.a
Descriptive data	14*	(a) Give characteristics of study participants (eg demographic, clinical, social) and information on exposures and potential confounders	Results
		(b) Indicate number of participants with missing data for each variable of interest	Table 1-4, Figure 1
		(c) *Cohort study*—Summarise follow-up time (eg, average and total amount)	Results - Outcome, Figure 1
Outcome data	15*	*Cohort study*—Report numbers of outcome events or summary measures over time	Results – Outcome, Figure 1
Main results	16	(*a*) Give unadjusted estimates and, if applicable, confounder-adjusted estimates and their precision (eg, 95% confidence interval). Make clear which confounders were adjusted for and why they were included	n.a
		(*b*) Report category boundaries when continuous variables were categorized	Results
		(*c*) If relevant, consider translating estimates of relative risk into absolute risk for a meaningful time period	n.a
Other analyses	17	Report other analyses done—eg analyses of subgroups and interactions, and sensitivity analyses	n.a
**Discussion**			
Key results	18	Summarise key results with reference to study objectives	Discussions
Limitations	19	Discuss limitations of the study, taking into account sources of potential bias or imprecision. Discuss both direction and magnitude of any potential bias	Discussions – Strengths and limitations
Interpretation	20	Give a cautious overall interpretation of results considering objectives, limitations, multiplicity of analyses, results from similar studies, and other relevant evidence	Discussion
Generalisability	21	Discuss the generalisability (external validity) of the study results	Discussion
**Other information**			
Funding	22	Give the source of funding and the role of the funders for the present study and, if applicable, for the original study on which the present article is based	Funding

*Give information separately for cases and controls in case-control studies and, if applicable, for exposed and unexposed groups in cohort and cross-sectional studies.

n.a. – not applicable

Note: An Explanation and Elaboration article discusses each checklist item and gives methodological background and published examples of transparent reporting. The STROBE checklist is best used in conjunction with this article (freely available on the Web sites of PLoS Medicine at http://www.plosmedicine.org/, Annals of Internal Medicine at http://www.annals.org/, and Epidemiology at http://www.epidem.com/). Information on the STROBE Initiative is available at www.strobe-statement.org.
